# Topical Treatment of Oral Mucositis in Cancer Patients: A Systematic Review of Randomized Clinical Trials

**DOI:** 10.31557/APJCP.2020.21.7.1851

**Published:** 2020-07

**Authors:** Geisa Sant Ana, Ana Gabriela Costa Normando, Isabela Porto de Toledo, Paula Elaine Diniz dos Reis, Eliete Neves Silva Guerra

**Affiliations:** 1 *Health Sciences Faculty, University of Brasília, Brasília, Brazil. *; 2 *Brazilian Centre of Evidence Based Research, University of Santa Catarina, Florianopolis, Brazil. *

**Keywords:** Topical intervention, chemotherapy, radiotherapy, oral mucositis, randomized controlled trial

## Abstract

**Background and Purpose:**

Evidence-based protocols of topical therapy for oral mucositis (OM) induced by chemoradiotherapy (CRT) are continuously established and updated. Thus, the present systematic review aims to evaluate the scientific literature in terms of effectiveness of topical treatment of OM in cancer patients undergoing CRT.

**Materials and Methods:**

This systematic review was based on the Preferred Reporting Items for Systematic Reviews and Meta-Analyses (PRISMA) Checklist. Randomized clinical trials were identified through electronic database searches on CINAHL, Cochrane Library, LILACS, Livivo, PubMed, SCOPUS, and Web of Science. Grey literature was also assessed on Google Scholar, Open Grey, and ProQuest. The risk of bias in the included studies was assessed by the Cochrane Collaboration Risk of Bias Tool.

**Results:**

Twenty-three randomized clinical trials (n=1169 patients) met the inclusion criteria. Twenty-three different topical agents were examined and categorized into five groups: analgesics (30.4%), natural agents (21.7%), other topical agents (21.7%), antimicrobial agents (17.4%), and growth factors (8.8%). Of the included studies, 50% presented a resolution of OM within 14 days. Topical natural agents yielded good results with average resolution time of 3–7 days. The included studies generally demonstrated that patients treated with mouthwashes presented superior benefits compared to the control, depending on OM severity.

**Conclusion:**

Topical agents effectively reduced the severity of OM lesions and pain intensity in patients receiving chemoradiotherapy, although the effects varied by agent type. However, the heterogeneity in the results of these topical intervention studies underscores the need for standardized clinical trial methodologies.

**Clinical Relevance:**

Topical agents were effective in patients with severe OM lesions receiving chemoradiotherapy and are a good alternative of home care in relation to pain control, reduction of inflammation and consequent improvement in quality of life.

## Introduction

Oral mucositis (OM) is one of the most prevalent adverse effect of head and neck radiotherapy (RT) and chemotherapy (CT) that is characterized by an inflammatory response of the oral cavity and oropharynx. OM affects 20–40% of patients receiving conventional CT, up to 80% of patients undergoing hematopoietic stem cell transplantation and receiving high doses of CT and almost all patients undergoing head and neck RT (Dodd et al., 2003; Miranzadeh et al., 2015; Sheibani et al., 2015; Lalla et al., 2014). Generally, patients undergoing CT experience some degree of oral discomfort within 5–10 days after treatment initiation (Nagarajan, 2015), while those undergoing RT usually develop OM within 1–2 weeks of treatment. Generally, OM causes great discomfort during eating, drinking, and speaking consequently resulting in weight loss and a decline in general health condition (Sahebjamee et al., 2015; Mogensen et al., 2017). 

Recent reports have described the complex pathogenic mechanisms of OM, which extends beyond immediate tissue damage to involve erythematous lesions that affect the entire epithelium, leading to severe ulceration, pain, submucosal hemorrhage, and infection. OM may interfere with antineoplastic treatment, leading to treatment interruption, a decreased quality of life, and compromised patient survival (Yoneda et al., 2007; Yen et al., 2012; Lalla et al., 2008; Raber-Durlacher et al., 2010; Ferreira et al., 2017; Trotti et al., 2003). Moreover, OM leads to a considerable economic burden, since it increases costs related to symptoms management, nutritional support, secondary infection treatment, and hospitalizations (Elting et al., 2007).

Currently, OM management mainly involves pain control, oral decontamination, inflammation reduction, oral hemorrhage management, and nutritional support (Lalla et al., 2014; Lalla et al., 2008). The Mucositis Study Group of the Multinational Cancer Support Care Association and the International Society of Oral Oncology (MASCC/ISOO) has proposed clinical practice guidelines for the management of OM that include palliative care and assumed future targeted therapeutic interventions (Lalla et al., 2014). Several studies have investigated alternative topical interventions that may reduce the symptoms and severity of OM, including allopurinol, benzydamine (Lalla et al., 2014; Tsavaris et al., 1991; Abbasi et al., 2007), chlorhexidine (Kin-Fong and Ka Tsui, 2006; Diaz-Sanches et al., 2015; Dodd et al., 2000), sucralfate (Dodd et al., 2003), diphenhydramine, morphine (Cerchietti et al., 2003), phenytoin (Baharvand et al., 2010; Baharvand et al., 2015), glutamine (Dodd et al., 2000; Anderson et al., 1989), aluminum hydroxide, palifermin, and propolis (Akhavankarbassi et al., 2016). Still, no evidence supports a standard systemic or topical therapy or preventive measure for OM induced by CT and/or RT. 

Taking into account that in many health services, patients do not have access to strategies for the prevention of OM, it is necessary that they have an alternative of home care in relation to pain control, reduction of inflammation and consequent improvement in quality of life. Given that topical agents are more easily applied, relatively inexpensive and have fewer side effects when compared to systemic therapies, the present systematic review aimed to summarize the scientific evidence available in the literature regarding the clinical practice of using topical agents as a therapeutic alternative for OM in patients undergoing cancer treatment.

## Materials and Methods

*Protocol and registration*

This systematic review was conducted according to the Preferred Reporting Items for Systematic Reviews and Meta-Analyses (PRISMA) Checklist (Moher et al., 2009; Shamseer et al., 2015). The protocol was registered in the International Prospective Register of Systematic Reviews (PROSPERO) database under registration number CRD42017073116 (Prospero, 2017).

*Study Design and terminology definition*

The present study is a systematic review of randomized controlled trials that assessed topical agents for OM treatment in cancer patients undergoing CT and/or RT. Topical intervention was defined as any treatment applied to the oral mucosa with local effects, including mouthwashes, creams, ointments, and jellies.

*Eligibility criteria*

*Inclusion criteria*

This systematic review followed the PICOS (population, intervention, comparison, outcome, and study design) approach in order to define the inclusion criteria. Only randomized clinical studies (S) assessing the effects of topical agents (I) for OM treatment (O) in cancer patients aged ≥18 years who underwent CT and/or RT (P). Any comparisons were considered for inclusion and only full-text articles were considered.

*Exclusion criteria*

The exclusion criteria were (1) patient age <18 years; (2) topical intervention for OM prevention; (3) non-randomized clinical trials; (4) reviews, letters, personal opinions, book chapters, and conference abstracts; (5) language restrictions; (6) full text unavailability; (7) studies with the same sample; and (8) use of hematopoietic stem cell transplantation as a treatment modality.

*Information sources and search strategy*

To identify literature published until April 17, 2019, individual search strategies were applied to the following electronic databases: CINAHL EBSCO, Cochrane Library, LILACS, Livivo, PubMed, SCOPUS, and Web of Science (Appendix 1). A gray literature search on Google Scholar, Open Grey, and ProQuest Dissertations and Theses Global was also performed. The references of included studies were manually screened for potential studies that could have been missed on database search. Duplicate references were removed using Rayyan, a reference manager software (Ouzzani et al., 2016). 

*Study selection*

During a two-phase study selection process, two authors (GSA and AGCN) independently reviewed the titles and abstracts of identified articles in Phase 1 and selected those appearing to meet the inclusion criteria. In Phase 2, these authors independently read the full texts of all selected articles and excluded those that did not meet the inclusion criteria (Appendix 2). Disagreements between evaluators were resolved by consensus, with final decisions by a third reviewer (IPT) if needed. 

*Data collection process*

One author (GSA) collected key data from each selected article, which were crosschecked for accuracy by a second reviewer (AGCN). Disagreements were resolved by discussion and mutual agreement among GSA, AGCN, and IPT. The following information was recorded for all included studies: author(s), publication year, country, patients’ ages (years), cancer type, cancer treatment, intervention type, control type, sample size (cases and controls), follow-up period, and main conclusions (Tables 1 and 2). 

*Risk of bias in individual studies*

The risk of bias of included trials was assessed by the Cochrane Risk of Bias (RoB) tool. “High,” “low,” or “unclear” risk scores were based on the randomization method; allocation concealment; blinding of participants, personnel, and outcome assessors; completeness of outcome data; and selective reporting (Higgins and Green, 2011). The reviewers compared evaluations, resolved disagreements and reported their RoB assessments using Review Manager software (RevMan 5.3, The Nordic Cochrane Centre, Copenhagen, Denmark). 

*Summary measures*

The primary outcome of this systematic review was a reduction in the OM severity grade based on the World Health Organization assessment scale. The secondary outcomes were the scores for erythema, wound healing, pain intensity, and eating and drinking ability. Any type of outcome measurement was considered in this review (categorical and continuous variables).

*Risk of bias across studies*

Individuals using novel topical interventions for OM management were compared with individuals using placebo and/or routine mouthwashes. Clinical heterogeneity (by comparing variability among the participant´s characteristics and outcomes assessed), methodological (by comparing the variability in study design and risk of bias), and statistical heterogeneity were considered in order to critically analyze the results. 

## Results

*Study selection*

**Figure 1:**
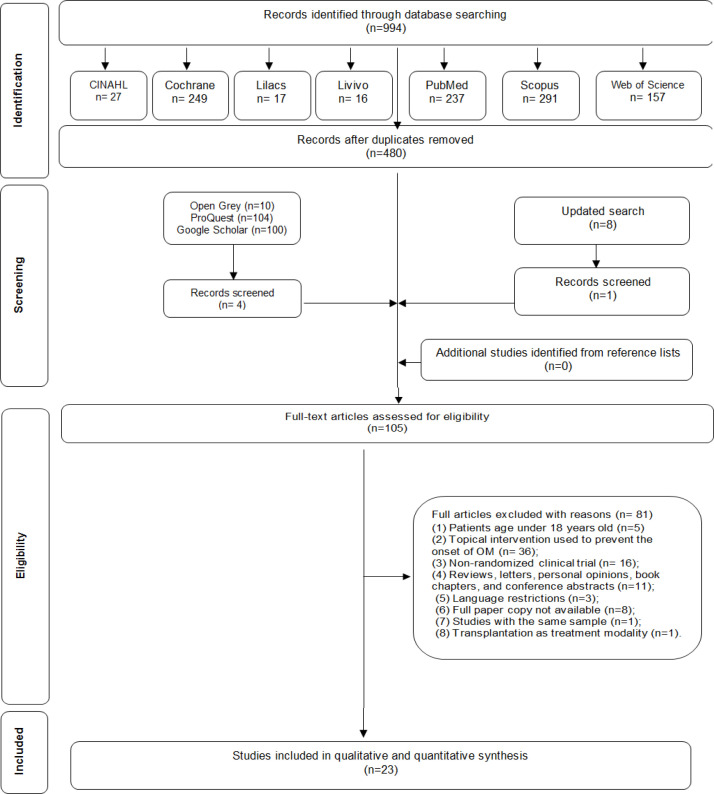
Flow Diagram of Literature Search and Selection Criteria Adapted from PRISMA

In phase 1, 994 citations were identified in seven electronic databases, and 480 remained after removing duplicates. Any references were included from gray literature. After screening the titles and abstracts, 376 references were excluded as irrelevant to the research question. One more reference was included after an updated search. A manual search of the reference lists yielded no additional studies. The full texts of 105 articles were screened (phase 2), and 81 were excluded (Appendix 2). Finally, 23 were selected for the descriptive analysis. A flow chart of the study identification, inclusion, and exclusion process is shown in Figure 1.

*Study characteristics*

 The 23 included studies (n=1,169 patients) were performed in 10 countries: Argentina (Cerchietti et al., 2003), Austria (Sprinzl et al., 2001; Hejna et al., 2001), China (Lin et al., 2015), Iran (Miranzadeh et al., 2015; Baharvand et al., 2016; Baharvand et al., 2015; Akhavankarbassi et al., 2016; Mansouri et al., 2016; Sarvizadeh et al., 2015), Italy (Porta et al., 1994), India (Satheeshkumar et al., 2010), Spaim (Cabrera-Jaime et al., 2018)Switzerland (Vayne-Bossert et al., 2010), Turkey (Erdem et al., 2014; Erden and Ipekcoban, 2017), Taiwan (Yen et al., 2012), and USA (Dodd et al., 2000; Dodd et al., 2003; Leenstra et al., 2014; Limaye et al., 2013; Rothwell etal., 1990; Wadleigh et a;., 1992). All articles described randomized clinical trials and were published in English during 1990–2017. The majority (47%) of the evaluated patients (552 patients) were diagnosed with head and neck cancer, followed by gastrointestinal, blood, breast, lymphatic, urinary tract, hepatocellular, and other/unknown cancers. The study sample sizes ranged from 9 (Vayne-Bossert et al., 2010) to 142 (Dodd et al., 2000). 

Twenty-two studies mentioned the follow-up duration (mean, 14 days; range, <1–45 days). Different topical interventions were classified as analgesics (30.4%), natural agents (21.7%), other topical agents (21.7%), antimicrobial agents (17.4%), and growth factors (8.8%). Here, intervention refers to the provided experimental treatments, while control refers to placebo and/or routine mouthwash. Table 1 summarizes the descriptive characteristics of studies assessing patients undergoing CT exclusively. Table 2 summarizes the descriptive characteristics of studies assessing patients undergoing chemoradiotherapy and RT. 

*Risk of bias within studies*

**Figure 2:**
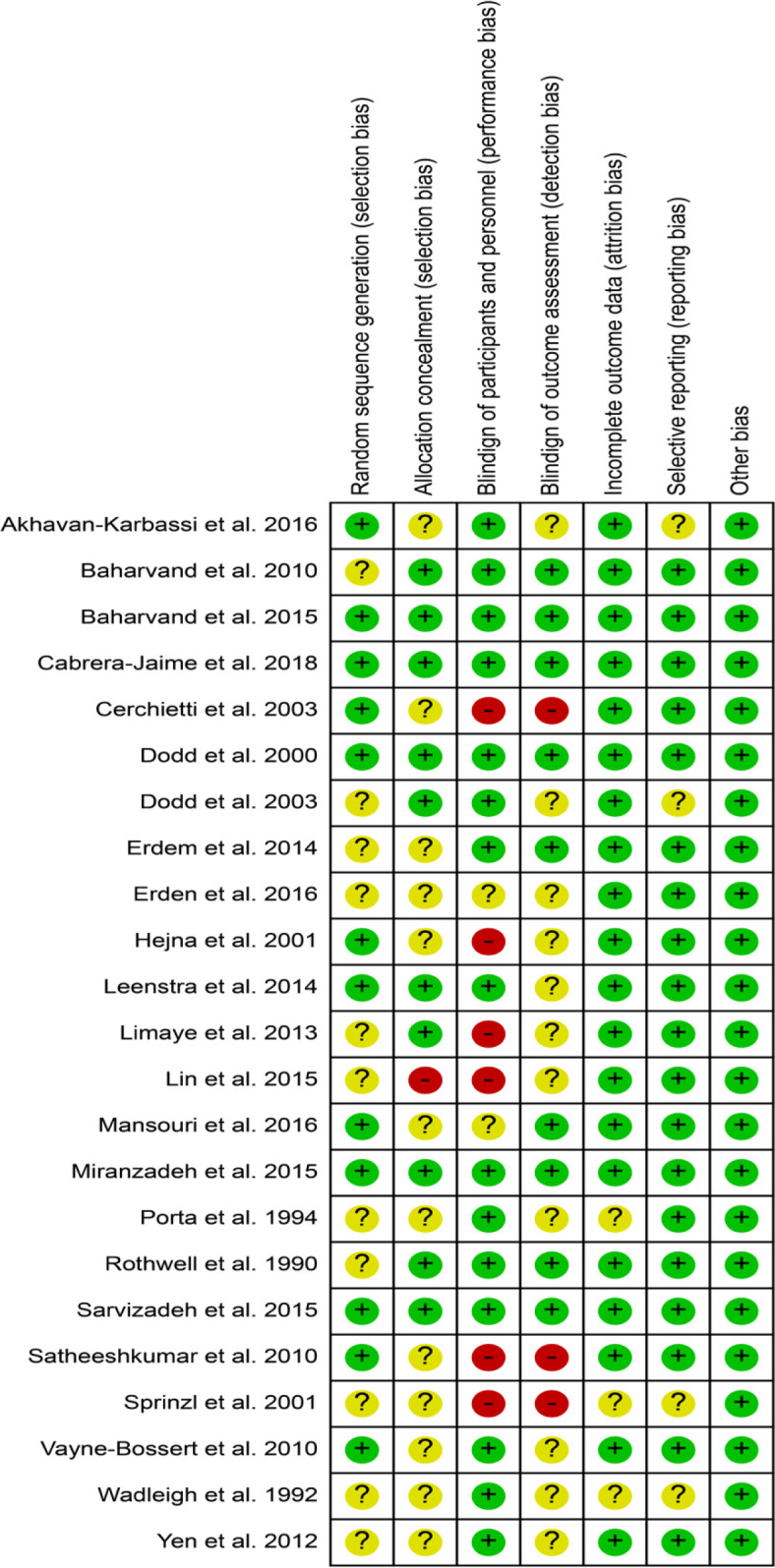
Risk of Bias Summary: review authors' judgements about each risk of bias item for each included study (+ = low; − = high;? = unclear).

Six studies had a high RoB due to one or more domains which compromised the reliability of the results (Cerchietti et al., 2003; Sprinzl et al., 2001; Lin et al., 2015; Satheeshkumar et al., 2010; Limaye et al., 2013; Hejna et al., 2001). Five studies had a low RoB in all evaluated domains (Miranzadeh et al., 2015; Baharvand et al., 2010; Baharvand et al., 2015; Sarvizadeh et al., 2015; Cabrera-Jaime et al., 2018) and could be considered more reliable studies (Figure 2). “Unclear risk,” defined as insufficient or missing data that difficult the assessment of the original study, occurred in the “random sequence generation” and “allocation concealment” domains of 11 (Dodd et al., 2003; Yen et al., 2012; Cerchietti et al., 2003; Sprinzl et al., 2001; Erden and Ipekcoban, 2017; Lin et al., 2015; Porta et al., 1994; Erdem et al., 2014; Limaye et al., 2013; Rothwell and Spektor, 1990; Wadleigh et al., 1992) and 12 studies (Yen et al., 2012; Cerchietti et al., 2003; Anderson et al., 1989; Sprinzl et al., 2001; Erden and Ipekcoban, 2017; Porta et al., 1994; Satheeshkumar et al., 2010; Vayne-Bossert et al., 2010; Erdem and Güngörmüş, 2014; Wadleigh et al., 1992; Hejna et al., 2001; Mansouri et al., 2012), respectively. “High risk” in the “blinding of participants and personnel” domain occurred in six studies (Cerchietti et al., 2003; Sprinzl et al., 2001; Lin et al., 2015; Satheeshkumar et al., 2010; Limaye et al., 2013; Hejna et al., 2001). Most studies (n=18, 78.3%) had a low RoB in the domains of “incomplete outcome data” and “selective reporting” (Miranzadeh et al., 2015; Yen et al., 2012; Cerchietti et al., 2003; Baharvand et al., 2015; Erden and Ipekcoban, 2017; Lin et al., 2015; Mansouri et al., 2016; Sarvizadeh et al., 2015; Porta et al., 1994; Satheeshkumar et al., 2010; Vayne-Bossert et al., 2010; Erdem et al., 2014; Leenstra et al., 2014; Limaye et al., 2013; Rothwell and Spektor, 1990; Hejna et al., 2001; Cabrera-Jaime et al., 2018; Cerchietti et al., 2002).

*Results of individual studies*

All 23 articles described different types of topical agents for OM treatment. Despite heterogeneity in the evaluated topical interventions, most patients receiving CT and/or RT exhibited reduced OM severity (i.e., grade) and/or pain intensity.

*Synthesis of results*

*Treatment characteristics*

The treatment characteristics are shown in Tables 1 and 2. More than half (n=63,2%) of the included patients received CT alone (Table 1), while almost 46% (n=537) received RT and/or CT (Table 2). Most studies of different cancer treatments identified the incidence of mucositis as a secondary outcome. Twelve studies evaluated the treatment of head and neck cancer, while 11 included several types of cancer (Tables 1 and 2). Of the 23 topical agents evaluated in this descriptive analysis, natural agents, analgesics, antimicrobial agents, growth factors, and others were applied to 209, 148, 98, 32, and 65 patients, respectively (Table 3). 

*Effects of interventions*

Studies on natural topical agents evaluated propolis (Akhavankarbassi et al., 2016), royal jelly (Erdem and Güngörmüş, 2014), Aloe vera gel (Mansouri et al., 2016), Achillea millefolium distillate (Miranzadeh et al., 2015), dioctahedral smectite and iodine glycerin (Lin eta l., 2015), and Plantago major extract (Cabrera-Jaime et al., 2018). These agents reduced OM intensity (grade 3) and pain within 4–14 days after the intervention (Miranzadeh et al., 2015; Akhavankarbassi et al., 2016; Lin eta l., 2015; Mansouri et al., 2016; Erdem and Güngörmüş, 2014). Natural topical agents, especially propolis (Akhavankarbassi et al., 2016) (n=20) and royal jelly (Erdem and Güngörmüş, 2014) (n=51), yielded superior results, with mean OM resolution times of 3–7 days (Table 4). Moreover, 65% of patients using propolis were completely healed by day 7, while 98% of those using royal jelly were completely healed in 3–4 days. Both treatments were administered as mouthwashes (Akhavankarbassi et al., 2016; Erdem and Güngörmüş, 2014). Like propolis, honey was associated with rapid recovery times and quicker healing than control treatments in patients with CT- and RT-induced mucositis (Aghamohammadi and Hosseinimehr, 2016; Tonkaboni et al., 2015; Maria et al., 2017; Zakaria, 2017). Honey also significantly reduced the severity of radiation-induced grade 3–4 mucositis (Amanat et al., 2017).

Topical analgesics are essential for pain control, and consequently for an appropriate food and fluid intake, communication, and sleep (Quinn et al., 2017). Studied topical analgesics included 0.5% or 1% phenytoin (Baharvand et al., 2010; Baharvand et al., 2015), 1% or 2% morphine (Cerchietti et al., 2003; Sarvizadeh et al., 2015; Vayne-Bossert et al., 2010), doxepin (Leenstra et al., 2014), and sucralfate (Dodd et al., 2003). Phenytoin mouthwash significantly improved patients’ pain and quality of life (Baharvand et al., 2010; Baharvand et al., 2015). Only one study on topical morphine described pain relief 28 minutes after the first use of mouthwash, with an average duration of relief of 216 minutes (Cerchietti et al., 2003). Doxepin rinse significantly reduced mouth and throat pain due to OM caused by RT and CT for HNC (P<0.001), however no significant correlation was found between this topical intervention and OM severity (Leenstra et al., 2014). Similarly, the use of topical sucralfate had no significant impact on OM severity (P=0.85) or pain reduction (P=0.54) (Dodd et al., 2003), suggesting the need for further randomized clinical trials with these agents. 

The studied topical antimicrobials included chlorhexidine gluconate (Dodd et al., 2000; Erden and Ipekcoban, 2017), nystatin + diphenhydramine + tetracycline + hydrocortisone (Rothwell and Spektor,1990), and triclosan (Satheeshkumar et al., 2010). Erden (2017) evaluated the efficacy of chlorhexidine on oral nutrition transition times in patients with CT-induced OM and observed a significant difference in days for OM resolution between the chlorhexidine (8.53±1.04) and control groups (13.53±1.69). In contrast, Dodd (2000) found no significant differences in the time of OM resolution (P=0.59) or in patients’ pain ratings over time among chlorhexidine and control mouthwashes groups. The use of oral rinse containing nystatin, diphenhydramine, tetracycline and hydrocortisone resulted in reduced OM severity compared to control group (Rothwell and Spektor,1990), as well as the use of triclosan was also capable of reducing the severity and duration of OM (Satheeshkumar et al., 2010).

Regarding growth factors, two studies on human granulocyte and macrophage colony-stimulating factor (GM-CSF) (Sprinzl et al., 2001; Hejna et al., 2001) yielded conflicting results. Hejna et al., (2001) recommended the topical use of GM-CSF for the treatment of CT-induced OM in patients with head and neck cancer since this topical treatment was effective on reducing the time of resolution of OM (P=0.0008) when compared to control. On the other hand, Sprinzl et al., (2001) did not recommend this application since there was no statistical difference between GM-CSF and conventional mouthwash in terms of OM severity. This difference may have probably occurred because in the study by Hejna et al., (2001) the patients were submitted to CT only, while in the study by Sprinzl et al., (2001) the patients were submitted to an association of CT and head and neck RT, causing a more severe mucositis.

Other topical agents included vitamin E oil (Wadleigh et al., 1992), allopurinol (Porta et al., 1994), AG013 (ActoBiotic) (Limaye et al., 2013), and 5% phenylbutyrate (Yen et al., 2012). Topical vitamin E oil, which has antioxidant effects, was reported by Wadleigh et al., (1992) that found 66% of patients receiving vitamin E intervention experienced a complete resolution of their lesions within 4 days of treatment initiation (median: 3 days), became asymptomatic, and were able to eat. The major pharmacological effects of allopurinol and its main metabolite, alloxanthin, involve the inhibition of xanthine oxidase (Porta et al., 1994; Fields et al., 1996). Porta et al., (1994) reported that all patients receiving CT developed grade 2–3 stomatitis, which resolved completely or partially by allopurinol mouthwash in 40.9% and 86.3% of patients, respectively. AG013 is an oral rinse containing the recombinant *L. lactis* strain engineered to secrete the mucosal protectant hTFF1. Limaye et al., (2013) reported that treatment with AG013 led to a 35% reduction in the mean duration of ulcerative OM (UOM) vs. placebo and reduced the numbers of unplanned office and emergency room visits. Furthermore, 29% of individuals receiving AG013 had none or 1 day of UOM; all other participants had ≥2 days of UOM. Yen et al., (2012) demonstrated that patients receiving a mouthwash containing phenylbutyrate and histone deacetylase inhibitor had significantly lower intensity of OM ulceration than those receiving a placebo (p=0.0485); suggesting that phenylbutyrate enhanced oral nutrition intake compared to the control (P=0.0085).

Twenty-one of 23 topical agents were administered as mouthwashes while one study used as a cream vehicle (Lin et al., 2015). Treatment with mouthwashes containing the following 15 agents were effective on reducing the duration of severe OM (functional impairment): propolis (Akhavankarbassi et al., 2016), royal jelly (Erdem and Güngörmüş, 2014), Aloe vera gel (Mansouri et al., 2016); Achillea millefolium distillate (Miranzadeh et al., 2015), 0.5% phenytoin (Baharvand et al., 2015), chlorhexidine gluconate (Dodd et al., 2000), chlorhexidine (Erden and Ipekcoban, 2017), nystatin+diphenhydramine+tetracycline and hydrocortisone, triclosan (Satheeshkumar et al., 2010), GM-CSF (Hejna et al., 2001), allopurinol (Porta et al., 1994), AG013 (Limaye et al., 2013), and 5% phenylbutyrate (Yen et al., 2012). 

*Risk of bias across studies*

The use of similar and robust methodologies in the included studies reduced the potential for misinterpretation. All included studies were randomized controlled trials and the majority were considered to be of moderate risk of bias, for these reasons, were considered to be relatively homogeneous in terms of methodological characteristics. When it comes to clinical aspects, the studies were considered similar in terms of participant characteristics and outcomes, but considerably heterogeneous in relation to topical interventions, consequently impacting the unfeasibility of a meta-analysis. Nevertheless, the results of our review could be considered consistent and trustworthy.

**Table 1:** Summary of the Descriptive Characteristics of Studies that Assessed Patients Undergoing Chemotherapy for Various Types of Cancers (n=11)

Study characteristics	Population characteristics						Intervention characteristics							
Author Year Country	Age Mean/range (years)		Cancer type		Cancer treatment		Intervention (# of cases)		Control (# of controls)		Follow-up (days)		Main conclusions	
Baharvand et al. 2010 Iran	C: 38.8±13.8 K: 33.3±14.8		Solid tumors (2) Blood malignancies (10)		Chemotherapy		0.5% Phenytoin mouthwash (6)		Placebo mouthwash (6)		14		Two weeks after study initiation, mucositis severity was significantly lower in the treatment group than in the control group. Lesions persisted for 4.5 days in the treatment group and 3–7 days in the control group. The minimum duration of lesion healing in the intervention group was 6 days, and 2 subjects required >14 days. The mucositis grade was determined using the WHO scale, and pain intensity was measured using an NRS.	
Cabrera-Jaime et al. 2018 Spain	59.5±14.3		Solid tumors		Chemotherapy		NaHCO3-Plantain (15)		NaHCO3- NaHCO3 (16) NaHCO3-Chlorhexidine (19)		7–14		There were no statistically significant differences in healing time or pain intensity among the three treatment groups. This study was the first to assess Plantago major for the treatment of OM in cancer patients. The intervention was not superior to sodium bicarbonate or chlorhexidine. However, a double dose of sodium bicarbonate (in mouthwash) was associated with a shorter healing time (5 vs. 7 days). This finding supports the use of alkaline oral care products as an evidence-based therapeutic approach to OM prevention and treatment and provides a focus for future research and care strategies. The mucositis grade was determined using the WHO scale, and pain intensity was measured using VAS.	
Dodd et al. 2000 USA	C: 59.24±14.89 K1: 59.47±14.27 K2: 57.39±14.62		Breast Colon Non-Hodgkin lymphoma Other		Chemotherapy		0.12% Chlorhexidine gluconate mouthwash (51)		“Magic” mouthwash (42) Salt and soda (49)		12		No significant differences in the time to OM sign and symptom resolution (P=0.59) or the patients’ pain ratings over time were observed among the three mouthwash groups. All three oral rinses appeared to provide no value other than the benefit of systematic oral hygiene protocol. Pain intensity was measured using an NRS. The study was limited by the lack of description of the mucositis grading scale.	
Erden et al. 2017 Turkey	≥18		Gastric Colon Pancreatic Rectal Metastatic cancer Unknown etiology		Chemotherapy		Chlorhexidine mouthwash (30)		Control group: cryotherapy (water mouthwash) (30)		21		There was a statistical difference in the moment of transition to oral nutrition for patients in the experimental groups. The time of oral nutrition time in the first experimental group that applied chlorhexidine was lower than in the group that applied cryotherapy and the control group (P<0.01). Chlorhexidine mouthwash is recommended for the treatment of OM. The mucositis grade was determined using the WHO scale. The study was limited by the lack of description of the pain measurement scale.	
Hejna et al. 2001 Austria	C: 58 (39–77) K: 73 (48–80)		Colorectal Gastric Pancreatic Breast Cholangiocellular		Chemotherapy		GM-CSF mouthwash (15)		AA mouthwash (16)		2.8 ± 0.7 (2–4)a		Topical GM-CSF treatment significantly reduced the duration and time to resolution of OM, including the pretreatment plus treatment periods (P=0.0008), as well as the duration of treatment until the complete remission of lesions (P<0.0001) when compared with AA. Topical GM-CSF may therefore be the treatment of choice for OM induced by standard cytotoxic chemotherapy. The mucositis grade was measured using the WHO scale. The study was limited by the lack of description of the pain measurement scale.	
Limaye et al. 2013 USA	C: 1x/day: 61(42–66) 3x/day: 54 (26–64) 6x/day: 52 (42–56) K: 54 (18–63)		Head and neck		Chemotherapy		AG013 (ActoBiotic) mouthwash (17) 1x/day (5) 3x/day (6) 6x/day (6)		Placebo mouthwash (8)		14		AG013 appeared to effectively reduce mucositis induced by PF (cisplatin, 5-fluorouracil) or TPF (docetaxel, cisplatin, 5-fluorouracil), but additional studies with larger sample sizes are recommended. Subjects treated with AG013 exhibited a 35% decrease in the mean percentage of days with UOM (WHO grade >2) vs. placebo. Furthermore, 29% of subjects who received AG013 had 0 or 1 day of UOM, whereas all subjects who received placebo experienced at least 2 days of UOM. The mucositis grade was determined using the WHO scale. The study was limited by the lack of description of the pain measurement scale.	
Lin et al. 2015 China	53.0 (19–78)		Lymphoma Breast Colorectal		Chemotherapy		DSIG (dioctahedral smectite and iodine glycerin) cream (63)		Topical mouthwash (gentamicin, saline and Vitamin B12) (67)		5		DSIG cream significantly reduced the duration of OM and relieved symptoms. A significant downward trend in mucosal lesions was observed in the DSIG cream subgroup vs. the topical buccal rinse group after 5 days of treatment. The DSIG cream also significantly reduced the OM repair time (4.68±0.98 vs. 8.76±1.80 days, P<0.001). The mucositis was measured using the OAG. The study was limited by the lack of description of the pain measurement scale.	
Mansouri et al. 2016 Iran	C: 46.25±18.17 K: 47.78±18.28	Acute myeloid leukemia Acute lymphocytic leukemia		Chemotherapy		Aloe vera mouthwash (32)		Routine mouthwash: normal saline, chlorhexidine, and nystatin (32)		3–14		The 2 groups differed significantly in terms of stomatitis intensity and pain between days 3 and 14 (P<0.05 and P=0.013, respectively), thus confirming the study hypothesis and demonstrating that Aloe vera could effectively reduce stomatitis intensity and pain. The mucositis grade was measured using the WHO scale. Pain intensity was measured using a VAS.	
Miranzadeh et al. 2015 Iran	C: 56.46±14.32 K: 55.54±14.01	Gastrointestinal Leukemia Lung Bone Kidney Breast		Chemotherapy		Achillea millefolium distillate mouthwash (28)		Routine mouthwash: lidocaine, dexamethasone, sucralfate, diphenhydramine solution (28)		14		The severity of OM was reduced on days 7 and 14 after the intervention, with only 3.6% and 0% of the experimental group exhibiting grade 3 or 4 OM, respectively, vs. more than 60% of the control group. A. millefolium distillate mouthwash reduced the severity of OM without side effects and could be used by patients during chemotherapy. The mucositis grade was measured using the WHO scale. The study was limited by a lack of description of the pain measurement scale.	
Porta et al. 1994 Italy	57.8 (30–68)	Colon Gastric Rectal		Chemotherapy		Allopurinol mouthwash (22)		Placebo mouthwash (22)		7		Allopurinol mouthwash completely or partially resolved OM in 40.9% and 45.4% of patients, respectively. OM persisted for an average of 4 days in the allopurinol group, vs. 7.5 days in the control group. Allopurinol appears to be simple and cost-effective. The study was limited by a lack of description of the scales used to measure the mucositis grade and pain.	
Wadleigh et al. 1992 USA	39–71 years	Head and neck Esophageal Hepatocellular Acute myelogenous leukemia		Chemotherapy		Vitamin E topical oil (9)		Placebo oil (coconut and soybean oils) (9)		5		Six of 9 patients in the vitamin E group achieved a complete resolution of their lesions within 4 days of initiating therapy (median: 3 days), whereas 8 of 9 patients receiving placebo did not achieve a complete resolution during the 5-day study period (P=0.025). The topical administration of vitamin E may be effective for the treatment of chemotherapy-induced mucositis. The mucositis grade was measured using WHO scale. Pain intensity was measured using a VAS.	

WHO, World Health Organization; NRS, numeric rating scale; C, case; K, control; VAS, visual analogue scale; OAG, Oral Assessment Guideline; OM, oral mucositis; GM-CSF, granulocyte-macrophage colony-stimulating factor; UOM, ulcerative oral mucositis.

**Table 2:** Summary of the Descriptive Characteristics of Studies that Assessed Patients Receiving Chemoradiotherapy and Radiotherapy for Head and Neck Cancer and Various Types of Cancer (n=12).

Study characteristics	Population characteristics			Intervention characteristics			
Author Year Country	Age Mean/range (years)	Cancer type	Cancer treatment	Intervention (# of cases)	Control (# of controls)	Follow-up (days)	Main Conclusions
Akhavan-Karbassi et al. 2016 Iran	≥18	Head and neck	Chemotherapy and radiotherapy	Propolis mouthwash (20)	Placebo mouthwash (sterile water with allowed additives) (20)	7	On day 7 of the trial, 65% of patients in the propolis group were completely healed. There were significant differences in the incidence of OM, wound, and erythema between the propolis and placebo groups, but no significant differences in eating and drinking abilities. Propolis-based mouth rinse is safe and effective for the treatment of RT-induced mucositis. The mucositis grade was determined using the WHO scale. The study was limited by a lack of description of the pain measurement scale.
Baharvand et al. 2015 Iran	C: 52.75±13.23 K: 56±14.65	Head and neck	Chemotherapy and radiotherapy	1% Phenytoin mouthwash (8)	Normal saline mouthwash (8)	21	The quality of life improved in both groups, but this outcome was significantly more obvious in the phenytoin group vs. the normal saline group (P<0.001). Although both groups achieved pain relief, it was more pronounced with phenytoin. Both groups experienced similar decreases in mucositis severity (P=0.154). The mucositis grade was determined using the WHO scale. Pain intensity was measured using an NRS.
Cerchietti et al. 2003 Argentina	First block: 56.9 (44–69) Second block: 55.6 (47–78)	Head and neck	Chemotherapy and radiotherapy	1% and 2% morphine mouthwash (First block: 10) (Second block: 22)	Water mouthwash (32)	First block: 1 (60 minutes) Second block: 1 (15, 30, 60, 120, 180 minutes)	A 2% morphine solution yielded better pain relief than a 1% solution (P=0.0238). Patients enrolled in the second block received a 2% morphine mouthwash, and the time to good or complete pain relief was 28 min after the first mouthwash, with an average duration of relief of 216 min. Topical morphine mouthwashes could be useful for alleviating painful chemoradiotherapy-induced stomatitis. The mucositis grade was measured using the WHO scale. Pain intensity was measured using an NRS.
Dodd et al. 2003 USA	C: 53.7 (18.1) K: 56.6 (13.0)	Head and neck	Chemotherapy and radiotherapy	Sucralfate mouthwash (14)	Salt + soda mouthwash (16)	30	The average worst severity ratings and average pain intensity scores did not differ significantly between the two mouthwash groups (P=0.85 and 0.54, respectively). Moreover, salt and soda are less expensive than micronized sucralfate. The study was limited by a lack of description of the scales used to measure the mucositis grade and pain intensity.
Erdem et al. 2014 Turkey	C: 53.8 (13.08) K: 50.69 (25.42)	Various types of malignancies	Chemotherapy and radiotherapy	Royal jelly (51)	Benzydamine hydrochloride and nystatin mouthwashes (52)	14	Royal jelly improved the signs and symptoms of OM and considerably reduced the time to healing, such that after 3–4 days all lesions had resolved in the jelly group, except for 1 case of grade 2 mucositis. The mucositis grade was determined using the WHO scale. The study was limited by a lack of description of the pain measurement scale and a lack of specification of the type(s) of cancer for which patients received treatment.
Leenstra et al. 2014 USA	C: 62 (39­–93) K: 60 (37­–86)	Head and neck	Chemotherapy and radiotherapy	Doxepin mouthwash (69)	Placebo mouthwash (71)	1 (5, 15, 30, 60, 120, 240 minutes)	Compared with placebo, doxepin yielded greater mean reductions in mouth and throat pain (-4.7 vs. -9.1; P<0.001). A doxepin rinse was significantly superior to placebo for treating OM pain due to RT ± chemotherapy for HNC. Further study is needed to fully elucidate this use of a doxepin rinse. The mucositis grade was determined using the WHO scale. Pain intensity was measured using an NRS.
Rothwell et al. 1990 USA	45–73	Head and neck	Radiotherapy	Test mouthwash (nystatin, diphenhydramine, tetracycline, and hydrocortisone) (5)	Cherry syrup containing sorbitol, magnesia and alumina suspension, and vitamins (7)	42	The topical application of nystatin, diphenhydramine, tetracycline, and hydrocortisone may reduce the incidence of RT-associated mucositis. Although the experimental group of patients developed mucositis, their symptoms were less severe and were not exacerbated beyond the third week of therapy. Pain intensity was measured on a scale of 0–5. The study was limited by a lack of description of the scale used to determine the mucositis grade.
Sarvizadeh et al. 2015 Iran	C: 47.5±14.6 K: 52.1±11.0	Head and neck	Chemotherapy and radiotherapy	2% Morphine mouthwash (15)	Magic mouthwash (magnesium aluminum hydroxide, viscous lidocaine, and diphenhydramine) (13)	6	Topical morphine effectively reduced the severity of OM in head and neck cancer patients. On day 6, a significant reduction in mucositis severity was observed in patients who received morphine, compared to those receiving the magic solution (P=0.045). Further studies with larger sample sizes and longer follow-ups are needed prior to the recommendation of routine topical morphine use. The mucositis grade was determined using the WHO scale. The study was limited by a lack of description of the pain measurement scale.
Satheeshkumar et al. 2010 India	C: 63.67±11.5 K: 65.9±12.9	Head and neck	Radiotherapy	Triclosan mouthwash (12)	Sodium bicarbonate mouthwash (12)	45	Triclosan may be effective for the management of RT-induced OM. There was no significant statistical difference between the intervention and control groups until the likelihood of progressing from grade 2–3 (P>0.05). Only one patient (8%) in the intervention group progressed to grade 4 mucositis, compared to 10 patients (83%) in the control group. A triclosan mouth rinse was superior to a sodium bicarbonate mouth rinse for the control of OM in terms of severity and duration. The mucositis grade was determined using the WHO scale. Pain intensity was measured using a VAS.
Sprinzl et al. 2001 Austria	C: 60 (49–82) K: 57 (42–75)	Head and neck	Chemotherapy and radiotherapy	GM-CSF mouthwash (Leukomax) (17)	Conventional mouthwash (pantocain, hydrocortisone acid, cional kreussler, and bepanthen) (18)	21	In a statistical analysis, GM-CSF was not superior to conventional mouthwash in terms of OM, pain perception, incidence of secondary infection, and abnormal hematological parameters. Therefore, topical GM-CSF is not recommended for the treatment of OM induced by chemoradiotherapy in patients with HNC. The mucositis grade was determined using the WHO scale. Pain intensity was measured using a VAS.
Vayne-Bossert et al. 2010 Switzerland	55.1±3.0	Head and neck	Chemotherapy and radiotherapy	2% Morphine mouthwash (4)	Placebo mouthwash (quinine diHCl) (5)	7	A morphine mouthwash yielded a mean (±SD) pain relief duration of 124±98 min vs. 126±81 minutes for placebo (P>0.01). It was not possible to conclude that local morphine via mouthwash can effectively treat local pain associated with OM. This result is distinct from the good peripheral analgesic effects of local opioids when applied to painful malignant and non-malignant skin ulcers. The mucositis grade was determined using the WHO scale. The study was limited by a lack of description of the pain measurement scale.
Yen et al. 2012 Taiwan	C: 51.1 (10.6) K: 54.8 (12.1)	Head and neck	Chemotherapy and radiotherapy (HNC)	5% Phenylbutyrate mouthwash (17)	Placebo mouthwash (same base as intervention) (19)	Patients began treatment after randomization and continued until 4 weeks after completion of RT	The intensity of ulceration in response to a cumulative RT dose of 6000–7000 cGy, which induced the most devastating phase of mucositis, was significantly lower in patients who received phenylbutyrate vs. those who received placebo (P=0.0485). Phenylbutyrate mouthwash appeared to significantly decrease the impact of OM in patients receiving RT or chemoradiotherapy for HNC. The mean duration of severe mucositis (WHO ≥ 3) was 2 days in the phenylbutyrate group and 12 days in the placebo group. The mucositis grade was determined using the WHO scale. The study was limited by a lack of description of the pain measurement scale.

WHO, World Health Organization; NRS, numeric rating scale; C, case; K, control; VAS, visual analogue scale; OM, oral mucositis; GM-CSF, granulocyte and macrophage colony-stimulating factor; RT, radiotherapy; HNC: head and neck cancer; SD, standard deviation

**Table 3:** Evidence of the Efficacies of Different Topical Interventions for Chemotherapy/Radiotherapy-Induced Oral Mucositis

Group	Topical intervention (n)	Objective	Oral mucositis model	Sex (% female)	Follow-up (days)	Most significant result	Proposed mechanism	Ref.
Topical natural agents	Propolis mouthwash (20)	This study aimed to determine the ability of propolis treatment to reduce the OM score, oral cavity erythema, and wound formation and to restore normal eating and drinking abilities in patients undergoing chemotherapy for head and neck carcinoma	Clinical studies of chemotherapy (methotrexate)-induced mucositis in patients with head and neck cancer	Not e stimated	7	All variables (erythema, wound formation, eating and drinking ability, and mucositis) improved significantly with propolis. Wound and OM scores decreased significantly in the placebo group. Interestingly, 65% of patients in the propolis group were completely healed at day 7 of the trial.	Anti-inflammatory, antibacterial, analgesic, collagen synthesis	Akhavan-Karbassi et al.
	Royal jelly mouthwash (51)	This study was conducted to evaluate the effect of royal jelly administrated via mouthwash on oral mucositis in patients undergoing radiotherapy and chemotherapy.	Clinical studies of chemoradiotherapy-induced mucositis in cancer patients	61%	4	Times to healing: 3–4 days for most grades in the royal jelly group vs 13–14 days for mucositis grades 2–3 in the control group	Anti-inflammatory, antioxidant, antibiotic	Erdem et al. 2014
	Aloe vera mouthwash (32)	The study aimed to evaluate the effectiveness of Aloe vera for reducing pain intensity and oral mucositis scores.	Clinical studies of chemotherapy-induced mucositis in patients with acute myeloid leukemia and acute lymphocytic leukemia	70%	3–14	The two groups differed significantly in terms of the intensity of stomatitis and pain between days 3 and 14 (P<0.05 and 0.013, respectively).	Antioxidant (polysaccharides, anthraquinone, lectin, superoxide dismutase and glycoprotein, amino acids, vitamins C and E and minerals)	Mansouri et al. 2016
	Achillea millefolium distillate mouthwash (28)	This study was designed to investigate the effect of A. millefolium distillate-containing solution on chemotherapy-induced oral mucositis.	Clinical studies of chemotherapy-induced mucositis in patients with gastrointestinal, lung, bone, kidney, breast, and blood (leukemia) cancers	57.10%	14	In the experimental group, the average healing time for OM grade 3 or 4 was 14 days. However, at this time, the rate of patients with OM grade 3 or 4 was increased to over 60% in the control group.	Anti-inflammatory, antimicrobial	Miranzadeh et al. 2015
	DSIG (dioctahedral smectite and iodine glycerin) cream (63)	This study aimed to compare the efficacy of DSIG cream with a topical mouth rinse (containing saline, gentamicin, and Vitamin B12) for treating chemotherapy-induced oral mucositis.	Clinical studies on chemotherapy-induced mucositis in patients with lymphoma, breast cancer, colorectal cancer	27.70%	5	In the experimental group, on day 5, 85.7% of patients had achieved complete regression of oral mucositis (P<0.001). However, only two patients (3.0%) obtained completed OM regression in the control group.	Naturally adsorbent DSIG (antimicrobial)	Lin et al. 2015
	Plantain (Plantago major extract) mouthwash (15)	This study aimed to evaluate the efficacy of a Plantago major extract mouthwash versus 0.12% chlorhexidine or 5% sodium bicarbonate (aqueous) for the treatment of oral mucositis symptoms in cancer patients with solid tumors.	Clinical studies on the symptomatic treatment of chemotherapy-induced oral mucositis in patients with solid malignancies.	Not estimated	14	Plantago major extract was no more beneficial than a sodium bicarbonate or chlorhexidine solution for the treatment of oral mucositis.	Anti-inflammatory	Cabrera-Jaime et al. 2018
Topical Analgesics	0.5% Phenytoin mouthwash (6)	This study aimed to compare a phenytoin mouthwash, an analgesic and wound-healing agent, with placebo for the treatment of chemotherapy-induced oral mucositis.	Clinical studies of chemotherapy-induced mucositis in solid tumors and blood malignancies.	58.30%	14	A minimum duration of 6 days was required for lesion healing in the experimental group. The proportion of patients with grade 2–3 oral mucositis was reduced to 0% after 1 week.	Analgesic and wound healing agent; “Phenytoin promotes wound healing by a number of mechanisms including stimulation of fibroblast proliferation, facilitation of collagen deposition by inhibiting the activity of collagenase enzymes, and antibacterial activity. Furthermore, by stabilizing neural fiber membranes and reducing the inflammatory response, phenytoin contributes to the topical pain relief.	Baharvand et al. 2010
	1% Phenytoin mouthwash (8)	This study aimed to investigate the effectiveness of a 1% phenytoin mouthwash in patients undergoing chemotherapy or radiotherapy for head and neck carcinoma.	Clinical studies of chemoradiotherapy-induced mucositis in various head and neck cancers, including oropharyngeal squamous cell carcinoma (SCC), tongue SCC, laryngeal SCC, mucoepidermoid carcinoma of the submandibular gland, and supraglottic SCC	Not estimated	21	Initially, seven patients presented with grade 2 mucositis; after 3 weeks, this number was reduced to four patients. The mucositis severity decreased in both groups, but this difference was not significant.	Analgesic and wound-healing agent; quality of life evaluation	Baharvand et al.2015
	1% and 2% Morphine mouthwash (first block: 10)	This study aimed to analyze the effect of a topical morphine mouthwash on damaged tissues in patients with head and neck cancer who developed mucositis induced by chemotherapy or radiation therapy.	Clinical studies on chemoradiotherapy-induced mucositis in patients with head and neck cancer	28.10%	First block: 1 (60 minutes) Second block: 1 (15, 30, 60, 120, 180 minutes)	After treatment (2% morphine mouthwash; second block), the mean duration of severe swallowing-related pain was 5.17±1.47 days, and the duration of severe functional impairment was 1.52±1.31 days. Results indicate that for patients with radiotherapy-induced stomatitis, morphine mouthwashes may be an effective and safe therapy to relieve pain and shorten the duration of functional impairment.	Systemic analgesic	Cerchietti et al. 2003
	2% Morphine mouthwash (15)	This study aimed to investigate the efficacy of topical morphine in comparison with a routine therapy (i.e., magic mouthwash) for the management of oral mucositis in patients with head and neck cancer.	Clinical studies of chemoradiotherapy-induced mucositis in patients with head and neck cancer	63.30%	6	On day 6, a significant reduction in mucositis severity was observed in patients who received morphine vs. those who received the magic solution (P=0.045).	Systemic analgesic; “Some evidence verified that opioid receptors are expressed on oral epithelial cells and morphine can accelerate the cell migration, which in turn can help to the wound healing process.”	Sarvizadeh et al. 2015
	2% Morphine mouthwash (4)	This study aimed to determine whether a morphine-containing mouthwash solution could decrease oral pain associated with radiotherapy- and/or chemotherapy-induced oral mucositis.	Clinical studies of chemoradiotherapy induced mucositis in patients with head and neck or breast cancer	77.80%	7	The symptom intensities did not differ statistically over the 6-day study period or between the two arms (analysis of variance).	Systemic analgesic (pain alleviation)	Vayne-Bossert et al. 2010
	Doxepin mouthwash (69)	This study aimed to test the efficacy of a doxepin oral rinse as an anesthetic/analgesic for oral mucositis pain caused by the treatment of head and neck cancer.	Clinical studies of chemoradiotherapy-induced mucositis in patients with head and neck cancer	19–21%	<1	In the second phase, the reported use of additional analgesia at the 2- and 4-hour time points did not differ between the doxepin and placebo arms.	Systemic analgesic	Leenstra et al. 2014
	Sucralfate mouthwash (14)	This study compared the efficacy of micronized sucralfate (Carafate R) mouthwash versus salt + soda mouthwash in terms of the severity of mucositis and mucositis-related pain the and time required for lesion healing in in patients with head and neck carcinoma who developed radiotherapy-induced mucositis.	Clinical studies of radiotherapy-induced mucositis in patients with head and neck cancer	30%	30	No significant differences in the average pain intensity scores were observed between the two mouthwash groups (t=0.63, P= 0.54).	Increased prostaglandin and mucus production, increased mucosal blood flow, increased growth factor binding due to sucralfate, a basic albumin salt of sucrose octasulfrate	Dodd et al. 2003
Topical antimicrobial	0.12% Chlorhexidine gluconate mouthwash (51)	This study analyzed the effectiveness of three mouthwashes used to treat chemotherapy-induced mucositis.	Clinical studies of chemotherapy-induced mucositis in patients with breast and colon cancer and non-Hodgkin lymphoma	64%	12	The three groups had similar times to the cessation of mucositis signs and symptoms (mean: 6.6–7.17 days).	Antimicrobial, anti-inflammatory	Dodd et al. 2000
	Chlorhexidine mouthwash (30)	This study aimed to compare the effects of chlorhexidine vs. a control on oral nutrition transition times in patients with chemotherapy-induced oral mucositis.	Clinical studies of chemotherapy-induced mucositis in patients with gastric, colon, pancreatic, rectal, and metastatic cancer (unknown cause)	50%	7–14	The mean transition time for oral nutrition differed significantly between the chlorhexidine group (8.53 ± 1.04 days) and the control groups (13.53 ± 1.69 days). This finding was statistically significant (P <0.05).	Antimicrobial, anti-inflammatory	Erden et al. 2016
	Nystatin, diphenhydramine, tetracycline, and hydrocortisone mouthwash (5)	This study aimed to analyze the effectiveness of an oral rinse comprising hydrocortisone, nystatin, tetracycline, and diphenhydramine for controlling radiation-related mucositis.	Clinical studies of radiotherapy-induced mucositis in patients with head and neck cancer	33%	42	As expected, the control group exhibited increasingly severe mucositis with increasing exposure to irradiation throughout the course of therapy. Mucositis severity increased in the experimental group during the first 3 weeks, but then decreased during the last 3 weeks of therapy.	Antimicrobial, anti-inflammatory	Rothwell et al. 1990
	Triclosan mouthwash (12)	This study aimed to determine the effectiveness of triclosan for the management of radiation-induced oral mucositis and to compare the effectiveness of a triclosan mouth rinse with that of a conventional sodium bicarbonate mouth rinse. Mucositis grade, body weight, food intake, and pain were assessed during weekly follow-ups throughout and after radiation treatment.	Clinical studies of radiotherapy-induced mucositis in patients with oral carcinoma	even distribution between men and women.	24	A triclosan mouth rinse was superior to a sodium bicarbonate mouth rinse for reducing the severity and duration of oral mucositis. The groups differed in terms of the recovery of mucositis from grade 3 to grade 0, which required a mean of 23.6 days in the intervention group vs. 36.5 days in the control group.	Antimicrobial, anti-inflammatory	Satheeshkumar et al. 2010
Topical growth factors	GM-CSF mouthwash (15)	This study aimed to analyze the efficacy of topical GM-CSF (molgramostim) vs. the combined topical use of an antiseptic agent (povidone-iodine) and amphotericin B (AA) in patients with chemotherapy-induced mucositis (World Health Organization; WHO) grades I–III.	Clinical studies of chemotherapy -induced mucositis in patients with head and neck cancer	54.80%	2–4	The ranges of therapy duration until complete remission of oral mucositis were 2–4 days in the GM-CSF group and 5–8 days in the AA group. Therefore, topical GM-CSF was recommended for the treatment of chemotherapy-induced oral mucositis in patients with head and neck cancer.	Growth factor activity	Hejna et al.2001
	GM-CSF mouthwash (Leukomax) (17)	This study compared GM-CSF with a conventional mouthwash (pantocain, hydrocortisone acid, cional kreussler, and bepanthen).	Clinical studies of chemoradiotherapy-induced mucositis in patients with advanced carcinoma (stage III–IV) of the oral cavity, oropharynx, and hypopharynx.	44%	21	GM-CSF was not superior to the conventional mouthwash in terms of oral mucositis, pain perception, the incidence of secondary infection, or hematological abnormalities. Therefore, topical GM-CSF was not recommended for the treatment of chemoradiotherapy-induced oral mucositis in patients with head and neck cancer	Growth factor activity	Sprinzl et al. 2001
Other topical agents	Vitamin E topical oil (9)	This study compared the efficacy of Vitamin E topical oil with that of a placebo oil (coconut and soybean oils).	Clinical studies of chemotherapy-induced mucositis in patients with head and neck, esophageal, and hepatocellular cancers and myelogenous leukemia	Not estimated	5	In the intervention group, 66% of patients experienced complete lesion resolution within 4 days of initiation (median: 3 days). Patients who responded to treatment became asymptomatic and were able to eat.	Antioxidant	Wadleigh et al. 1990
	Allopurinol mouthwash (22)	This study analyzed the efficacy of an allopurinol mouthwash for the treatment of 5-fluorouracil-induced stomatitis.	Clinical studies of chemotherapy-induced mucositis in patients with colon, gastric, and rectal cancers	Not estimated	4	Allopurinol mouthwashes resolved stomatitis completely in 40.9% of patients, with responses seen in 86.3%. The duration of oral mucositis was 4 days in the allopurinol group vs. 7 days in the control group.	Enzyme inhibition	Porta et al. 1994
	AG013 (ActoBiotic) mouthwash (17)	This study evaluated the safety and tolerability of orally applied AG013 at three daily dosages.	Clinical studies of chemotherapy -induced mucositis in patients with head and neck cancer	24%	30	AG013 reduced the mean percentage of days with ulcerative oral mucositis by 35%, compared to the placebo, and reduced the number of unplanned office and emergency room visits. Moreover, 29% of subjects who received AG013 had 0–1 day of ulcerative oral mucositis, compared to at least 2 days overall.	Biotherapeutic activity	Limaye et al. 2013
	5% Phenylbutyrate mouthwash (17)	This study evaluated the safety and efficacy of a 5% phenylbutyrate mouthwash used to mitigate oral mucositis during radiation therapy or concurrent chemoradiotherapy in patients with head and neck cancer.	Clinical studies of chemoradiotherapy-induced mucositis in patients with oral cavity carcinoma, nasopharyngeal carcinoma, oropharyngeal carcinoma, and hypopharyngeal carcinoma	35%	21	During the most devastating phase of mucositis (radiotherapy), the intensity of ulceration was significantly lower in patients receiving phenylbutyrate mouthwash vs. those receiving placebo (P=0.0485). Patients treated with phenylbutyrate were more likely to retain the ability to intake food orally vs. controls (9.0% vs. 3.8%, P=0.0085, chi-square test).	Histone deacetylase (HDAC) inhibitor	Yen et al. 2012

GM-CSF, granulocyte-macrophage colony-stimulating factor; OM, oral mucositis;

**Table 4.** Mucositis frequency and time for healing by cancer treatment and topical intervention agent

Cancer treatment	Topical intervention (n)	Cancer type	Time to healing (days)	References
Chemoradiotherapy	Royal jelly mouthwash (51)	Various types of malignancies	3–4	Erdem et al. 2014
Chemotherapy	Aloe vera mouthwash (32)	Acute myeloid leukemia, acute lymphocytic leukemia	3–14	Mansouri et al. 2016
Chemotherapy	DSIG (dioctahedral smectite and iodine glycerin) cream (63)	Lymphoma, breast cancer, colorectal cancer	5	Lin et al. 2015
Chemotherapy	Allopurinol mouthwash (22)	Colon, gastric, rectal cancers	5	Porta et al. 1994
Chemotherapy	Vitamin E topical oil (9)	Head and neck, esophageal, and hepatocellular cancers; acute myelogenous leukemia	5	Wadleigh et al.1992
Chemoradiotherapy	1–2% Morphine mouthwash (51)	Head and neck cancer	6–7	Sarvizadeh et al. 2015; Cerchietti et al. 2003; Vayne-Bossert et al. 2010
Chemoradiotherapy	Propolis mouthwash (20)	Head and neck cancer	7	Akhavan-Karbassi et al. 2016
Chemotherapy	NaHCO3-plantain (15)	Solid tumors	7–14	Cabrera-Jaime et al. 2018
Chemotherapy	0.12% Chlorhexidine gluconate mouthwash (51)	Breast, colon, and other cancers; non-Hodgkin lymphoma	12	Dodd et al. 2000
Chemotherapy	Achillea millefolium distillate mouthwash (28)	Gastrointestinal leukemia; lung, bone, kidney, and breast cancers	14	Miranzadeh et al. 2015
Chemotherapy	0.5% Phenytoin mouthwash (6)	Solid tumors, blood malignancies	14	Baharvand et al. 2010
Chemotherapy	AG013 (ActoBiotic) mouthwash (17)	Head and neck	14	Limaye et al. 2013

* Including only topical interventions associated with resolutions ≤14 days.

## Discussion

*Summary of evidence*

Cancer is one of the most common causes of death worldwide and its incidence has been gradually increasing, mainly due to both aging and growth of the population, as well as changes in the prevalence and distribution of the main risk factors for cancer (Bray et al., 2018). Its treatment depends on several factors that include the type of the tumor, the location, the clinical and pathological staging as well as the patient’s health status. Currently, there are several types of CT and RT that can be used alone or in combination to manage the disease. Both therapies are extremely effective in destroying tumor cells but as a result they end up causing side effects so damaging that treatment often needs to be interrupted. One of the most prevalent side effects is oral mucositis, which affects around 40% of patients undergoing chemotherapy, such as Methotrexate, Cisplatine and 5-Fluorouracil, and almost 100% of patients undergoing head and neck RT (Sonis, 2009; Scully et al., 2003).

The pathobiology of oral mucositis is divided into 5 phases: initiation, signaling, amplification, ulceration and healing. Once the chemotherapeutic drug or radiotherapy contacts the mucosa, several chemical changes occur in the tissue, resulting in the release of reactive oxygen species that in turn activate transcription factors capable of amplifying the production and release of inflammatory cytokines. This amplification causes a cycle of constant production of cytokines that result in clinically evident and painful ulceration susceptible to bacterial colonization and secondary infection (Sonis, 2009). Thus, the need of early intervention is fundamental in order to reduce the severity of the injury. Although many therapeutic agents have been investigated, no effective prevention or treatment standard protocol has been completely successful to handle OM (Dos Santos Filho et al., 2018). 

It is not uncommon in clinical dentistry practice for patients to ask for medications that they can apply at home in order to reduce pain and control inflammation. Prevention with photobiomodulation has been widely accepted and applied, but, unfortunately, in many health services such therapy is still inaccessible to many patients (Zadik et al., 2019). Thus, topical therapeutic alternatives for OM are necessary, which are cost-effective, easily applicable and cause less additional side effects in patients who are already systemically compromised.

For the best of our knowledge, this is the first systematic review of randomized clinical trials that compiled the highest level of scientific evidence available in the literature in terms of efficacy of topical agents for OM in patients with cancer. The included studies generally demonstrated that patients treated with mouthwash presented superior benefits when compared to the control, depending on mucositis severity. 

In the case of natural agents, royal jelly treatment was effective during the initial but not final stages of OM, and the corresponding control group benefited from benzydamine hydrochloride and nystatin mouthwash (Erdem and Güngörmüş, 2014). Moreover, propolis mouthwash improved oral health in patients undergoing CT (Akhavankarbassi et al., 2016), thus reinforcing the recommendations for therapeutic mouthwashes to promote oral hygiene, prevent/treat infections, moisten the oral cavity, and provide pain relief (Quinn et al., 2017). Both honey and propolis exert various anti-inflammatory effects, antioxidant activity, prostaglandin synthesis-inhibiting activity in mucosal tissue, pro-immune effects via the stimulation of phagocytic activity and cellular immunity, and healing effects in epithelial tissues. Propolis is rich in iron and zinc, which are important elements in collagen synthesis (Akhavankarbassi et al., 2016; Erdem and Güngörmüş, 2014; Zakaria, 2017). The anti-inflammatory agents Achillea millefolium distillate (Miranzadeh et al., 2015) and Plantago major extract (Cabrera-Jaime et al., 2018) yielded different responses. Achillea millefolium mouthwash improved the mean healing time of grade 3–4 OM to 14 days, whereas Plantago major extract was not superior to control treatment (sodium bicarbonate or chlorhexidine). However, Plantago major extract reduced the healing time from 7 to 5 days when combined with sodium bicarbonate in a mouthwash. Accordingly, strategies involving oral hygiene products are evidence-based therapeutic approaches to mucositis prevention and treatment (Cabrera-Jaime et al., 2018). The antioxidant activity of topical Aloe vera gel is mediated by polysaccharides, anthraquinone, lectin, superoxide dismutase, glycoproteins, amino acids, vitamins C and E, and minerals. Mansouri et al., (2016) reported significantly reduced pain and OM intensity between 3 and 14 days after the use of Aloe vera mouthwash (p<0.05 and 0.013, respectively). 

Among topical analgesics, phenytoin mouthwash yielded significant improvements in pain and quality of life (Baharvand et al., 2010; Baharvand et al., 2015). The topical antimicrobial chlorhexidine exhibited activity against gram-positive and gram-negative bacteria and fungi and had minimal systemic adverse reactions when used at a low concentration, which reduced absorption in the gastrointestinal tract (Dodd et al., 2000; Erden and Ipekcoban, 2017). 

GM-CSF is a hematopoietic growth factor that promotes neutrophil proliferation and differentiation. Previously, Liang et al., (2017) reported that GM-CSF could prevent and treat CT- and RT-induced OM in patients with head and neck cancer. In our review, two studies reported conflicting results regarding the efficacy of topical GM-CSF for OM (Sprinzl et al., 2001; Hejna et al., 2001), although this discrepancy might have been related to the use of RT. Specifically, RT-induced OM begins with inflammation of the oral mucosa, tongue, and pharynx, followed by a normal tissue lesion for 7–98 days (Limaye et al., 2013; Maria et al., 2017; Sonis, 2010). 

In addition to the primary outcome of the present review, which was to assess the effect of topical therapies currently available on OM control, some studies have contemplated other secondary outcomes. Nine included studies discussed the importance of oral hygiene, monitoring and controlling of opportunistic infections via antimicrobial treatments and preventive dental protocols, including selective extractions, restorations, and fluoride programs. These randomized controlled trials addressed the reduction of the incidence of sepsis in patients with OM, a considerable risk factor reported in most studies (Dodd et al., 2003; Akhavankarbassi et al., 2016; Sprinzl et al., 2001; Erden and Ipekcoban,2017; Mansouri et al., 2016; Sarvizadeh et al., 2015; Satheeshkumar et al., 2010; Rothwell and Spektor, 1990; Hejna et al., 2001). 

*Limitations*

This review had some limitations that should be considered. First, the methodological quality was overall moderate, mainly due to heterogeneity of the studies as a consequence of the large number of topical interventions. Second, there was also heterogeneity in terms of presentation of results among the studies, as some analyzed treatment evolution according to OM severity while others presented results with medians. Moreover, there was a wide variation on duration of the interventions, ranging from 1 day to 4 weeks. Due to all this considerable heterogeneity among reviewed studies, a meta-analysis could not be conducted. The absence of pain measurement scales was also a limitation. 

In conclusion, in this review, the efficacy of topical agents for OM in cancer patients undergoing CT and/or RT was evaluated. Particularly, topical natural agents yielded good results and significant improvements in the patients’ quality of life. Generally, topical agents reduced the OM severity and pain intensity in patients receiving CT and RT, although the effects varied among interventions. However, the heterogeneity of the studies’ results demonstrates the need to standardize the validated assessment instruments and similar interventions that would enable comparisons and analyses of treatment effects based on well-designed randomized clinical trials. 

## Abbreviations

OM, oral mucositis; CT, chemotherapy; RT, radiotherapy; RoB, risk of bias; GM-CSF, granulocyte and macrophage colony-stimulating factor; UOM, ulcerative oral mucositis.
